# Comprehensive Time-Series Analysis of the Gene Expression Profile in a Susceptible Cultivar of Tree Tomato (*Solanum betaceum*) During the Infection of *Phytophthora betacei*

**DOI:** 10.3389/fpls.2021.730251

**Published:** 2021-10-21

**Authors:** Daniel Bautista, Natalia Guayazan-Palacios, Maria Camila Buitrago, Martha Cardenas, David Botero, Jorge Duitama, Adriana J. Bernal, Silvia Restrepo

**Affiliations:** ^1^Department of Biological Sciences, Universidad de los Andes, Bogotá, Colombia; ^2^Department of Biology, University of Washington, Seattle, WA, United States; ^3^Department of Chemical and Food Engineering, Universidad de los Andes, Bogotá, Colombia; ^4^Department of Systems and Computing Engineering, Universidad de los Andes, Bogotá, Colombia

**Keywords:** *Phytophthora betacei*, plant–pathogen interaction, plant response, *Solanum betaceum*, time series analysis

## Abstract

*Solanum betaceum* is a tree from the Andean region bearing edible fruits, considered an exotic export. Although there has been renewed interest in its commercialization, sustainability, and disease management have been limiting factors. *Phytophthora betacei* is a recently described species that causes late blight in *S. betaceum*. There is no general study of the response of *S. betaceum*, particularly, in the changes in expression of pathogenesis-related genes. In this manuscript we present a comprehensive RNA-seq time-series study of the plant response to the infection of *P. betacei*. Following six time points of infection, the differentially expressed genes (DEGs) involved in the defense by the plant were contextualized in a sequential manner. We documented 5,628 DEGs across all time-points. From 6 to 24 h post-inoculation, we highlighted DEGs involved in the recognition of the pathogen by the likely activation of pattern-triggered immunity (PTI) genes. We also describe the possible effect of the pathogen effectors in the host during the effector-triggered response. Finally, we reveal genes related to the susceptible outcome of the interaction caused by the onset of necrotrophy and the sharp transcriptional changes as a response to the pathogen. This is the first report of the transcriptome of the tree tomato in response to the newly described pathogen *P. betacei.*

## Introduction

*Solanum betaceum*, commonly known as tree tomato or tamarillo, is a tree native from the Andean region where it is widely cultivated because it bears edible fruits (Castaño Monsalve et al., 2015). Phylogenetic analysis places *S. betaceum* in the monophyletic clade Cyphomandra, showing little divergence to its wild relatives (Bohs, 2007). Known for its vitamin content and antioxidant activity, *S. betaceum* could be considered a potential exotic export, demonstrated by the growing production of the crop in New Zealand and some Mediterranean countries (Acosta-Quezada et al., 2012). Despite this, crop sustainability is a limiting factor in its commercialization, particularly in disease management. Limited research has been done regarding cultivars with a well characterized pathogen resistance (Revelo et al., 2011; Castaño Monsalve et al., 2015).

*Phytophthora* species are described as some of the most prevalent pathogens of the tree tomato (Ramírez-Gil et al., 2017). These filamentous oomycetes present a wide host range in *Solanum* species, affecting relevant food crops such as potato and tomato. *Phytophthora infestans* and *Phytophthora betacei* have been described as the causal agents of late blight in *S. betaceum* (Chañag-Miramag et al., 2017; Ramírez-Gil et al., 2017; Mideros et al., 2018). Recent assessment of the *S. betaceum* and *P. betacei* interaction revealed a single large population of *P. betacei* dispersed throughout the Andean region, presenting different levels of susceptibility for common tree tomato cultivars (Chaves et al., 2020; Mideros et al., 2020).

The hemibiotrophic lifestyle of *Phytophthora* species allows the pathogen to present an initial biotrophic cycle, in which the infection is established aided by specialized structures called haustoria. Then there is a colonization phase that is followed by a switch to necrotrophy in which extensive tissue damage and sporulation are produced within a few days (Jupe et al., 2013). This method of infection requires an intricate interaction aimed at the inhibition and restraint of the plant response, which is achieved by the pathogen by releasing intracellular and apoplastic molecules called effectors (Fry, 2008). These effectors are capable of: (i) changing the expression of defense-related genes by modifying host histone acetylation (Kong et al., 2017), (ii) increasing the production of biomolecules needed by the pathogen (Breen et al., 2017), and (iii) orchestrating mechanisms to overcome the recognition of the host resistance strategies (Jing et al., 2016; Toruño et al., 2016).

Furthermore, with the aim to comprehend a plant–pathogen interaction, it is key to elucidate the plant immunity response to the pathogen. In the context of a dynamic molecular coevolution, plant immunity relies on the recognition of the pathogen to initiate a complex network of signal transduction to block the infection mechanisms of the pathogen (Fawke et al., 2015). The first line of defense involves the recognition of conserved pathogen-associated molecular patterns (PAMPs) by transmembrane pattern recognition receptors (PRRs), prompting pattern-triggered immunity (PTI). This is characterized by activation of salicylic acid signaling, cell wall strengthening, and production of phytoalexins, reactive oxygen species (ROS) and antimicrobial proteins that try to promote infection (Bigeard et al., 2015; Qi et al., 2017). Nonetheless, pathogen effectors suppress PTI to promote infection on the host, producing effector-triggered susceptibility (ETS). Thus, plants might overcome this strategy by detecting these effectors proteins with *R*-genes, generating effector-triggered immunity (ETI) that often results in hypersensitive response (HR) avoiding pathogen colonization (Ali et al., 2014; Neu et al., 2019).

The recent discovery of a new species of *Phytophthora*, *P. betacei* (Mideros et al., 2018), represents a great opportunity to depict for the first time the response of *S. betaceum* to the infection caused by the pathogen. Although the biological mechanism in which *Phytophthora* species cause infection has been studied in other *Solanum* species (Rodewald and Trognitz, 2013; Jiang N. et al., 2020; Witek et al., 2021), the response of *S. betaceum* to *P. betacei* remains unexplored (Acosta-Quezada et al., 2012). In this study, we characterized the expression profiles of pathogen-induced genes after the infection with *P. betacei* throughout different time points, describing the transcriptional events that mark plant immune response of a susceptible cultivar.

## Experimental Procedures

### Plant and Pathogen Material

The strain N9035 from the *P. betacei* collection at the Universidad de los Andes museum, maintained at 18°C in tree tomato agar medium (1.8% bacteriological agar, 1.8% sucrose, 0.05% CaCO_3_, and 20% tree tomato juice) was selected for inoculation. Susceptible *S. betaceum* plants belonging to the “Común” accession were obtained from certified seeds (Impulsemillas, Bogotá, Colombia). All seeds were submerged in distilled water for 24 h before germination following the manufacturer’s instructions. Then, the seeds were sown in peat to induce germination. Finally, seeds were transplanted into individual pots where germination and growth were carried out under greenhouse conditions (12 h light period, 18°C, 80–100% RH). All subsequent experiments performed in this study were done on 8–10-week old tree tomato plants.

### Inoculation of *Phytophthora betacei*

A sporangial suspension of *P. betacei* consisting of 3.5 × 10^5^ sporangia mL^–1^ (Mideros et al., 2018) was prepared using a hemocytometer. The suspension was inoculated on 21 plants during daytime (between 6:00 and 9:00 a.m.) as follows: three leaves of the same plant were drop-inoculated on the abaxial side using four 20 μL droplets of the adjusted suspension (two droplets on each side of the main vein). Subsequently, inoculated plants were placed inside a growth chamber (Percival Scientific Inc., Perry, IA, United States) at 17 ± 2°C, 80% relative humidity, and 12 h light period.

### Expression Profile of *Solanum betaceum*

#### Library Preparation and RNA Sequencing

In order to identify plant differentially expressed genes (DEGs) involved in the response to *P. betacei* infection along the infection cycle, RNA-seq analysis was performed on leaf tissue harvested from inoculated plants at 6, 12, 18, 24, 72, and 96 h post infection (hpi), as well as from uninoculated plant material, referred to as 0 hpi. For the RNA extractions, tissue was harvested from three plants at the indicated time points using a cork borer. In detail, tissue was collected from each inoculated leaf on the same plant (two leaf punches per inoculated leaf, six leaf punches total) and pooled together to make one biological replicate. Total RNA was obtained from each biological replicate using the RNeasy Plant Mini Kit^®^ (Qiagen, Germantown, MD, United States) according to the manufacturer’s instructions. The purified RNA was treated with DNase I, and its integrity and yield were measured using a 2100 Bioanalyzer (Agilent, Waldbronn, Germany).

#### Bioinformatic Analyses

All raw reads (∼1342 M) were screened for quality control using FASTQC v.0.11.2 (Babraham Bioinformatics, Cambridge, United Kingdom). Reads that presented adapter sequences, a length less than 36 bp, and bases with Phred quality score below 5 were trimmed with Trim Galore! (Martin, 2011; MacManes, 2014). Given that both plant and pathogen were present in the reads, a genome of *P. betacei* was used to remove pathogen transcripts (GCA_011320135.1), using BBDuk (BBMap- Bushnell B. – sourceforge.net/projects/bbmap/). In order to improve the assembly of the transcriptome of *S. betaceum*, possible sequencing mistakes were removed based on unique *k*-mers, using Rcorrector (Song and Florea, 2015) with default parameters. Contamination of the sequences was assessed performing a screening against UniVec database^1^ and *P. infestans* genome (ASM1229517v1) using Seal (BBMap- Bushnell B. – sourceforge.net/projects/bbmap/).

#### Transcriptome Assembly

To downsample the counts of the filtered reads, the software Trinity was used to perform an *in silico* normalization with a maximum coverage set to 30× (Grabherr et al., 2011). *De novo* and reference-based transcriptome assemblies were compared for further analyses. Using the normalized reads from all the time points, the *de novo* assembly was performed using Trinity 2.8.4 (Grabherr et al., 2011) and rnaSPAdes (Bushmanova et al., 2019). Among the 2,940,929,728 processed reads, 17,785,308 were used in the assembly after normalization, with a targeted 30× coverage. Because a completely *de novo* assembly approach was generating more transcripts with multiple isoforms than expected and low median length (N50) values, we performed a reference-guided assembly using a close species with a high quality reference genome. A genome guided transcriptome assembly based on potato (*Solanum tuberosum SolTub_3.0*), a closely related solanaceous crop with a high-quality genome, was also obtained by aligning the filtered reads with STAR (Dobin et al., 2013). We used StringTie (Pertea et al., 2015) and Trinity 2.8.4 to assemble the transcriptome with default settings.

#### Quality Assessment of *de novo Solanum betaceum* Transcriptome

Summary statistics of the assembly were obtained by running QUAST (Gurevich et al., 2013) with the previously mentioned files. To assert the completeness of the assembly, a BUSCO (Seppey et al., 2019) analysis with the “Solanaceae odb10” dataset was performed only with the longest isoform for Trinity generated assemblies and the hard filtered fasta for rnaSPAdes. DOGMA (Dohmen et al., 2016), a software with similar approach but focused on broad domain representation was also evaluated for each assembly with the previously mentioned files.

#### Estimation of Gene and Transcript Abundance and Expression Analysis

Non-normalized filtered reads from all time-series were aligned back to *de novo* and genome guided *S. betaceum* transcriptomes to estimate the abundance of transcripts using kallisto pseudoalignment (Bray et al., 2016) with default settings. The quantification was performed on the assembled transcripts and Trinity genes, which are related transcript sequences that share *k*-mers. The estimation matrix was later converted to transcripts per million (TPM) for sample normalization. To account for cross sample normalization, the reads were transformed by the trimmed mean values (TMM) approach (Robinson and Oshlack, 2010) and Log2 converted to run the clustering analysis. The principal component analysis (PCA) and heatmaps from the TMM counts were produced with the aid of Trinity software. Transcripts with TMM ≥ 1 for all replicates for each sample were selected. The differential expression analysis was performed with EdgeR with default settings (Robinson et al., 2010). Transcripts with a Log-fold change ≥ 4 and adjusted *P* < 0.001 (Benjamini–Hochberg procedure) were selected for further analysis.

### Downstream Analysis

#### Annotation of the Transcriptome

Coding sequences were predicted for the assembled transcripts with TransDecoder.^2^ A Blastp (parameters per default) search against the Swiss-Prot database (Bairoch and Apweiler, 2000) was performed for transcripts with predicted open reading frames, and a Blastx (parameters per default) for all the assembled transcripts, both with an *e*-value threshold of 10^–5^. The predicted proteins were also scanned for the identification of protein domains against the Pfam database using HMMER (Potter et al., 2018). In order to predict the presence of signal peptide cleavage sites, a search was submitted to SignalP 4.1 standalone software version (Nielsen, 2017). The results were loaded into a single SQLite database with the help of Trinotate (Bryant et al., 2017). The Gene Ontology (GO) terms associated with the matches from the annotation report were used in the downstream analysis. KEGG accession codes were retrieved and visualized with the help of The KEGG Automatic Annotation Server (KAAS) (Moriya et al., 2007).

#### Clustering Approaches

Expression profiles were assessed by a PCA based on abundance counts from all samples. In terms of differential expression, the TMM counts generated were used to analyze transcripts with a similar expression pattern throughout the infection time. Cluster analysis was performed *via* the software DPGP (McDowell et al., 2018) using maximum *a posteriori* estimation to partition the transcripts in 1,000 iterations. The resulting clusters were set out for enrichment analysis using TopGO (Alexa and Rahnenführer, 2009) and the differences in GO terms were evaluated in each cluster against all DEGs. Genes belonging to the clusters were annotated for KEGG pathways using KAAS. The heatmap was generated with the ComplexHeatmap package (Gu et al., 2016) in R software (v. 3.4.0), transforming the TMM count to Log2 and splitting the genes in groups according to the previously estimated clusters. Hierarchical cluster analysis was also performed to compare against the maximum *a posteriori* method.

### RT-qPCR Validation

We performed a RT-qPCR validation for four genes with different peaks of expression based on RNA-seq data. Primers were designed using the transcripts with primer-BLAST. Samples collected from the same plants used for RNA sequencing were used for RT-qPCR. RNA was extracted using InviTrap Spin Plant RNA Mini Kit (Invitek) following the manufacturer’s protocol. cDNA was synthetized using cDNA Reverse transcription Kit (Applied Biosystems, Foster City, CA, United States). The purified RNA was treated with DNase I, and its integrity and yield were measured using a 2100 Bioanalyzer (Agilent, Waldbronn, Germany). qPCR was performed to analyze the expression using Maxima SYBR Green qPCR (Thermo Scientific, Waltham, MA, United States) and a 7500-fast thermocycler (Applied Biosystems, Foster City, CA, United States). Pfaffl method was used to analyze data, using primer efficiency, *Elongation Factor* as housekeeping gene and 0 hpi as sample control (Pfaffl, 2001).

## Results

### Transcriptome Assembly and Catalog of Gene Expression in *Solanum betaceum* Across the Infection Course

**FIGURE 1:**
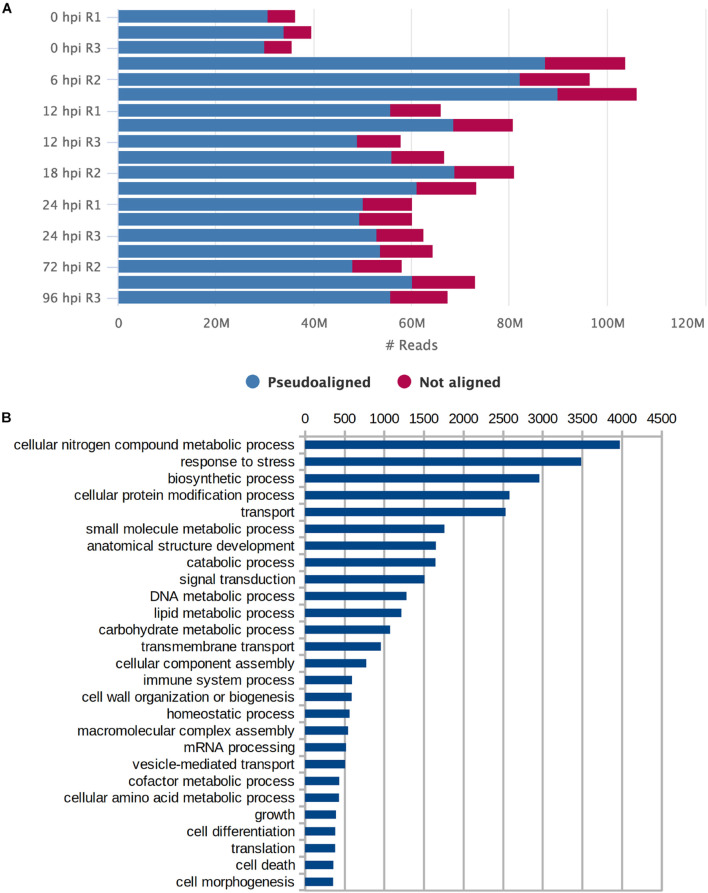
Assessment of transcriptome assembly. **(A)** Number of reads (in millions) per sample that pseudoaligned and not aligned to the transcriptome. **(B)** Gene Ontology terms for Biological Processes that were present in at least 2% of all transcripts.

We performed sequencing of mRNA transcripts of *S. betaceum* at six different time points after the infection with *P. betacei* to document the gene expression profile across the progression of the disease. Time points 6, 12, 18, 24, 72, and 96 were chosen to cover all transcriptomic regulation in the early time of biotrophy (6–24 h) and the switch to necrotrophy (72 and 96 h), according to Guayazán et al. (2017). Uninoculated samples were used as a control (referred here as time zero) (Supplementary Figure 1). Using the genome of *S. tuberosum* (*SolTub_3.0*) as reference to guide the assembly, the resulting transcriptome consisted of 46.1 Mbp, with 77,117 predicted transcripts (Table 1). An overall percentage of 83.98% of the reads could be aligned back to the assembled transcripts (Figure 1A). We found that for GO terms present in at least 2% of all transcripts, Biological Processes related to Cellular Nitrogen Compound Metabolic Process, Response to Stress, and Biosynthetic Process were the most abundant (Figure 1B). To evaluate the completeness of the transcriptome we searched for genes and domains conserved across *Solanaceae* species using the tools BUSCO (Gurevich et al., 2013) and DOGMA (Dohmen et al., 2016). We found intact orthologs of 2,440 (79.9%) conserved genes and 3,792 (95.10%) conserved domains (Table 1).

**TABLE 1:** Transcriptome assembly statistics for *Solanum betaceum* using *Solanum tuberosum* genome as reference.

Metrics	All samples
**Sequence**	
Unprocessed reads	2,940,929,728
Processed reads	2,487,875,164
Processed reads (normalized)	314,049,162
**Assembly**	
Total assembled bases	416,120,984
Total Trinity “genes”	62,375
Total Trinity “transcripts”	77,117
GC%	40.55
**Based on all transcripts**	
Contig N50	1420
Median contig length	386
Average contig	774.29
**Only longest isoform**	
Contig N50	1301
Median contig length	351
Average contig	704.45
**BUSCO**	
Complete BUSCOs	2440 (79%)
Missing	338 (11%)
Total BUSCOs group searched	3052
**DOGMA**	
Found/expected	3792/3872
Completeness (%)	97.3

*Metrics were generated by QUAST, BUSCO, and DOGMA for quality assessment of the transcriptome.*

**FIGURE 2:**
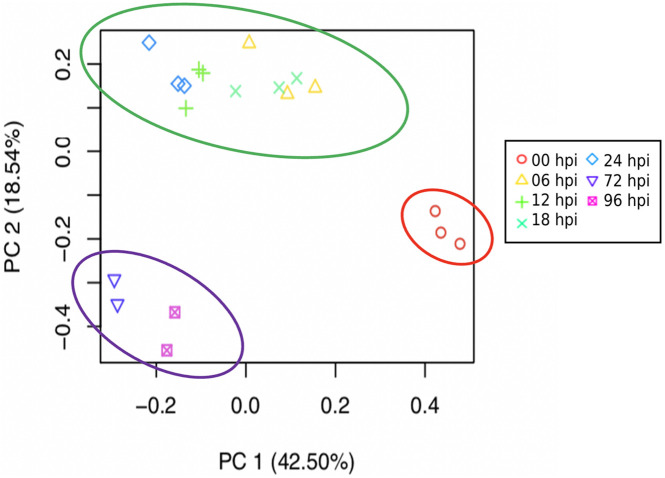
Principal component analysis (PCA) based on expression profile of the different times of infection (0, 6, 12, 18, 24, 72, and 96 h post-inoculated) shows a marked separation of the early and late stages of response of *S. betaceum* to *P. betacei*. Based on the correlation matrix for all sample replicates, each point represents a sample, and replicates share the same symbol. The circles highlight the two distinguishing groups that match a potential division between the biotrophic and necrotrophic stages: in green, an early biotrophic response stage is suggested. Necrotrophic late response is shown circled in purple.

Aligned read counts normalized as TMM were used to estimate and compare expression levels for the assembled transcripts across samples. Figure 2 shows a PCA illustrating the distances between samples based on TMM values, and taking into account the first two principal components. These components explain 61% of the variation. Most of the samples grouped its replicates compactly, with the exception of the third replicate at 6 and 24 hpi. Figure 2 also shows how grouping arises for certain conditions: the uninoculated samples were visibly separated from the rest, while the replicas belonging to the first hours of infection (6, 8, 12, and 24) were grouped and contrasted with the remaining conditions of 72 and 96 h.

### Clustering Based on Gene Expression Reveals Differentially Expressed Genes Related to Response to Hemibiotrophic Lifestyle

**FIGURE 3:**
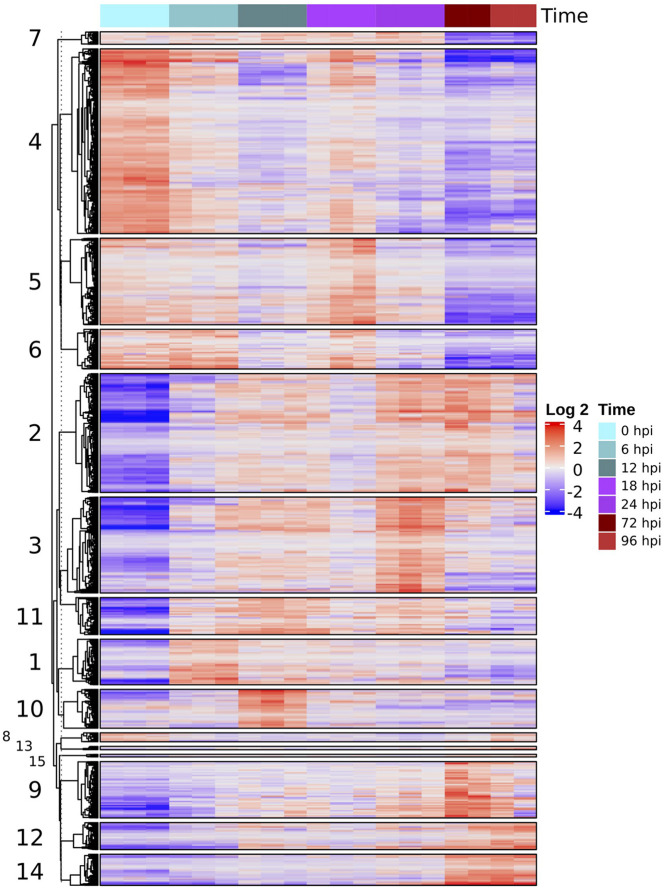
Heatmap of differentially expressed genes (four LCF, *P* < 0.001) based on normalized TMM counts compared against all samples were hierarchically clustered. The heatmap is divided into groups with similar expression profiles throughout the time series. The numbers of each group correspond to the number of each cluster in Figure 4.

We found 5,628 DEGs across all times (*P* < 0.001, four Log-fold change). Hierarchical clustering of these genes grouped similar expression profiles, most involving an activation of the host response to the pathogen (Figure 3). Groups 4, 5, 6, and 7 contained 2,306 genes that were downregulated during the infection relative to the control samples. GO terms enrichment analysis shows that most of these downregulated genes correspond to photosynthesis and secondary metabolism (Supplementary Figure 2).

In contrast, another set of genes were differentially expressed after inoculation with peaks of upregulation at different times during infection. Although there is some decrease in expression, specifically at the later stages, this pattern shows a marked regulation of transcription in response to the interaction with the pathogen. Most of these upregulated genes, belonging to groups 1, 2, 3, 10, and 11 (2,397 total) were related to plant defense, like the synthesis of terpenoid and phenylpropanoid, cell signaling, and pathogenesis related (PR) proteins (Supplementary Figure 2).

The remaining set of genes, groups 9, 12, and 14, appear to display a marked overexpression pattern in later stages (72 and 96 hpi) of infection. GO enrichment analysis showed an upregulation of biological process such as signaling (GO:0023052), leaf senescence (GO:0010150), different kinds of response to biotic stress (GO:0006950), and external stimuli (GO:0009607) (Supplementary Figure 3).

### Description of Differentially Expressed Genes Throughout the Progression of the Disease

**FIGURE 4:**
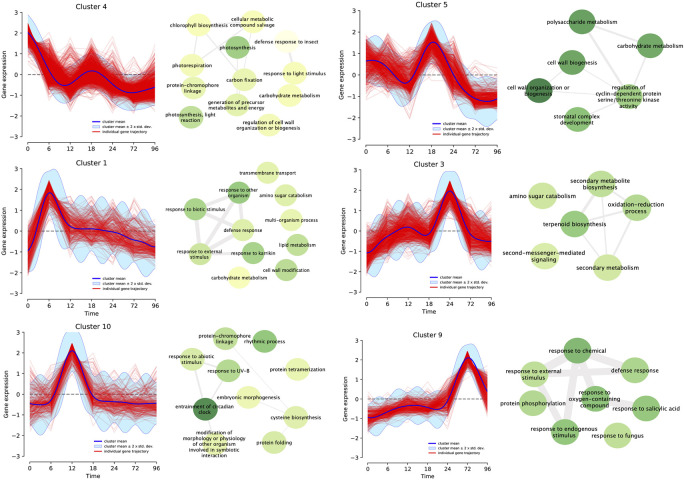
Differentially expressed genes (fourfold, *P* < 0.001) for all infection times (0, 6, 12, 18, 24, 72, and 96 h post-inoculation) were clustered based on their expression profile. Fifteen different clusters based on maximum *a posteriori* method were produced, and matched the number of each group in the heatmap. GO terms enrichment analysis was performed for each cluster. Connecting lines between GO terms indicates relation and darker green means higher *P*-value. Here we highlighted six of these clusters which demonstrate a single peak of overexpression of differentially expressed genes for each time point with their correspondent enriched GO Terms.

We performed a clustering analysis based on the trajectory of each gene across the time series to group genes that share similar expression profiles, obtaining 15 different clusters (Supplementary Figure 3). We performed an enrichment analysis of biological processes and KEGG pathway annotations, focusing on six clusters having a single highest expression peak in the time series (Figure 4). The next paragraphs describe the main results of this analysis in sequential order relative to the expression peak.

Cluster 4 includes genes that were overexpressed before the inoculation of the pathogen (0 hpi) and, as the infection progressed, turned off. Most of the enriched terms were related to photosynthesis (GO:0015979, GO:0019684, GO:0009765, and GO:0009853), regulation of plant cell wall (GO:1903338 and GO:0009827), and carbohydrate metabolism (GO:0015977 and GO:0005975). This is the largest cluster, with 1,317 genes accounting for 23% of all the DEGs. Belonging to this category, there were putative genes that code for pentatricopeptide repeat-containing proteins (PPR), the production of auxin, basic helix-loop-helix (bHLH) transcription factors, and leucine-rich repeat (LRR) receptor-like serine/threonine-protein kinase FLS2 (Supplementary Table 1). Plant-related pathways for this cluster included photosynthesis (20 genes), plant hormone signal transduction (8 genes) and plant–pathogen interaction (3 genes).

Cluster 1 with 325 genes showed an upregulation of expression 6 h post-inoculation. The most common enrichment GO terms were related to response to external biotic stimulus (GO:0043207), defense to other organism (GO:0098542), response to fungus (GO:0050832), cell wall modification (GO:0042545), carbohydrate and amino sugar metabolism (GO:0046348 and GO:0005975). *Ethylene response*, *WRKY* transcription factors and LRR receptor-like kinases are also present in this set. In terms of annotated KEGG pathways, amino sugar and nucleotide sugar metabolism were present while MAPK signaling pathway encompassed the abscisic acid receptor PYR/PYL family as a stress response.

Cluster 10 showed a peak of expression at 12 h. A total of 277 genes belonged to this cluster, which contains enriched GO terms related to modification of morphology or physiology of other organism involved in symbiotic interaction (GO:0044003), entrainment of circadian clock (GO:0009649), rhythmic process (GO:0048511), and response to abiotic stimuli (GO:0009628). Included in this set were *Pathogenesis-related protein PR-1* and *Cytochrome P450* monooxygenases. Genes belonging to Flavonoid biosynthesis (00941), Plant hormone signal transduction (04075) pathways were also described for this cluster.

Cluster 5 comprised 617 genes that shared the highest point of expression at 18 hpi. Enrichment GO terms for this set of genes were carbohydrate and polysaccharide metabolism (GO:0044262 and GO:000597), cell wall biogenesis or organization (GO:0071554, GO:0071555, and GO:0044036), stomatal complex development (GO:0010103) and regulation of cyclin dependent protein serine/threonine kinase activity (GO:1904029). Pathways related to this set were starch and sucrose metabolism, phenylpropanoid biosynthesis, plant hormone signal transduction (04075), and plant–pathogen interaction (04626). There were putative disease resistance proteins (RPP13-like protein 3, RGA3) as well as pathogenesis-related thaumatin-like protein.

Cluster 3 has 684 genes (11%) which showed an overexpression at 24 h post-inoculation. Most enriched GO terms were related to secondary metabolism: terpenoid biosynthesis (GO:0016114 and GO:0006721), secondary metabolism biosynthesis (GO:0044550 and GO:0019748), oxidation-reduction process (GO:0055114), amino sugar catabolism (GO:0046348), as well as second messenger mediated signaling (GO:0044550). Pyruvate metabolism, phenylpropanoid biosynthesis, amino sugar and nucleotide sugar metabolism, oxidative phosphorylation, MAPK signaling pathway–plant, plant hormone signal transduction, and plant–pathogen interaction were some of the pathways present in this subset. *WRKY*, *ethylene response transcription factors*, *basic form of pathogenesis-related protein 1*, and *putative late blight resistance protein homolog R1A-10* were some of the genes related to plant–pathogen interaction.

There were 402 genes in Cluster 9 that were activated at 72 hpi. There were enriched GO terms associated to general response to the pathogen: defense response (GO:0006952), response to fungus (GO:0009620), to endogenous stimulus (GO:0009719), to salicylic acid (GO:0009751), abscisic acid (GO:0009737), oxygen-containing compound, and to external stimulus (GO:1901701). Plant–pathogen interaction pathway showed five genes related to PAMP triggered immune response, MAPK signaling pathway included four genes. Transcription Factors like *WRKYs*, *ethylene response* and *mitogen-activated protein kinase 4* (*MPK4*) were also present.

### Catalogue of Resistance and Susceptible Genes Against *Phytophthora betacei*

Based on the annotation of transcriptome, we searched for resistance and susceptible terms. We focused on the transcripts which presented an expression of TMM ≥ 1. In terms of resistance genes related to the late blight, *putative late blight resistance protein homologs R1A-10*, *R1B-23*, *R1B-14*, and *R1C-3*, shows upregulation in three different time points: 6, 24, and 72 hpi (Supplementary Table 2). There were also genes coding for disease resistance proteins like *RPM1*, *RPP13*, *RPP8*, *LAZ5*, *Pik-1*, and putative disease resistance proteins such as *RGA1* and *RGA3* that were activated in those times. Pathogenesis-related proteins present in the clusters were: pathogen-related protein, pathogenesis-related protein STH-2, and thaumatin-like protein. They displayed expression at 6, 18, 24, and 72 h post-inoculation.

Clusters 2, 3, and 9 contain genes related to the plant–pathogen interaction pathway, expressing at 24 and 72 h post-inoculation. Genes present in this set were *elicitor-responsive protein 1* (*ERP*), *LRR receptor-like serine/threonine-protein kinase* (*FLS2*), *calcium-dependent protein kinase* (*CPK*), *pathogenesis-related protein 1* (*PR1*), *MPK4*, and *WRKY transcription factor 29*.

Susceptibility genes were also expressed throughout the time series, *DLO2*, *PMR5*, *protein-tyrosine-phosphatase MKP1*, and sugar transporters *SWEET1*, *SWEET2a*, and *SWEET12* stood out. Their expression profile shows an overexpression at the beginning of the infection (0 hpi) and at 18 hpi. Other susceptible genes like *enhanced disease resistance* 2 (*EDR2*), *protein-tyrosine-phosphatase* (*MKP1*), and *cellulose synthase A catalytic subunit 8* (*CeSa8*) were regularly expressed in all time-series (TMM ≥ 1).

### RT-qPCR Validation

Expression patterns were similar between RT-qPCR and RNA-seq data (Supplementary Figure 4). All the assessed genes were upregulated consistently with RNA-seq profile with a fairly strong positive relationship (Supplementary Figure 5). *Sb-O-PRK1* was inducted at 6 hpi, *Sb-TR-HY5* and *Sb-O-WSD1* were highly expressed at 6 and 12 hpi, *Sb-ERF098* expression was increased at 6 and 24 hpi and finally, *Sb-NRT1-PTR* was upregulated at 72 and 96 hpi. Altogether, these results support the RNA-seq time-serial pattern of gene expression.

## Discussion

### Assembly of the Defense Response Transcriptome of *Solanum betaceum*

We provide the first transcriptome assembly for tree tomato (*S. betaceum)* with a comprehensive expression profile across infection with *P. betacei*, one of the causal agents of late blight, a predominant disease that affects this crop (Ramírez-Gil et al., 2017). To achieve this goal, deep RNA sequencing was performed which allowed us to obtain a clear picture of plant gene expression against the pathogen. Initial attempts at *de novo* assembly of *S. betaceum* using Illumina paired-end reads generated a noisy transcriptome in which numerous unsupported transcripts were present. With the lack of a reference genome, we opted for a genome-guided assembly using the closest high-quality genome with the most mapped reads, which was *S. tuberosum* (*SolTub_3.0*). Metrics utilized to assess the quality of the assembly and its completeness such as BUSCO and DOGMA, demonstrates the usefulness of a well annotated genome of a close species as a reference to increase the quality of a *de novo* transcriptome assembly. Downstream analysis of expression values estimated from aligned read counts indicate that the transcripts assembled in this study were useful to follow the progression of the disease. Even though the number of aligned reads and the percentage of conserved genes captured by the assembled transcriptome were relatively high, the assembly of a high quality reference genome for *S. betaceum* is an urgent next step to enhance the study of disease resistance and other important traits at the molecular level. The sequencing resources provided in this work should be helpful to perform an accurate annotation of gene models and transcripts on any upcoming genome assembly.

### Host Transcription Expression Is Related With Hemibiotrophic Life Strategy of the Pathogen

Taking into account the biology of the hemibiotrophic pathogen *P. betacei*, we focused on characterizing the transcriptional changes by a susceptible host in response to a pathogen in an undescribed pathosystem. We observed, based on the expression profiles in all infection times (0, 6, 12, 18, 24, 72, and 96 h post-inoculation), the consistent clustering of the samples alongside pathogen infection, suggesting an active transcriptional interplay by host and pathogen. The variance of the transcriptional profile between before inoculation at 0 h, and all post infection times (6–96 hpi) (42.5%) indicates the induction of an intricate transcriptional response against the pathogen that differentiates through time.

This change of expression starts with the recognition of the pathogen by the plant, as DEGs comparing 0 hpi to the early hours of infection (6, 12, 18, and 24 hpi) included GO terms related to defense response. Oomycete elicitors, most likely being conserved PAMPs, could cause an immune response, inducing the expression of PAMP-triggered immunity (PTI) associated response genes (Fawke et al., 2015). We found expression of genes like *pathogenesis-related protein* 1, 4-B SHT-2, PTI-5, and *WRKY* transcription factor 29, linked to the production of phytoalexins, which are toxic to the pathogen (Rogers et al., 1996; Bigeard et al., 2015). Given that the cultivar employed is susceptible, the interaction between the plant and pathogen is compatible, and it is likely that the pathogen is successfully using effectors that halt a complete immune response (Duan et al., 2020).

We observed differences between the composition of enriched GO terms in early (6, 12, 18, and 24 hpi) and later hours (72 and 96 hpi) of the times series which denotes a disparity in transcription related to the pathogen. This result agrees with the biology described for this pathogen, as *P. betacei* has shown to switch from biotrophy to necrotrophic phase around 72 hpi (Guayazán et al., 2017). Similar global transcriptional changes in the host caused by *Phytophthora* pathogens have been described but have not been associated with the pathogen life cycle as we observed in this study (Gao et al., 2013; Evangelisti et al., 2017). GO terms enriched at 72 and 96 hpi included mainly defense response and cell death terms, while there is a general shutting off of regulation processes and signaling. This is consistent with the response to necrotrophic pathogens (Glazebrook, 2005; Guayazán et al., 2017).

### Time Series Analysis Explains the Patterns of Gene Expression Under Biotic Stress

To examine the transcriptional changes that are happening at each point of the time series, we clustered DEGs and focused on those that showed a clear single upregulation peak in a single time point. Regarding the genes with high expression before inoculation, two clusters showed general downregulation after inoculation, in GO terms related to photosynthesis (at 6 and 12 hpi). Most of these genes are chloroplast related genes that the plant could be shutting down as PAMPs are recognized in the early times of infection (Nomura et al., 2012). Although induction of defense responses requires energy, decrease of photosynthesis activity is explained as a defense mechanism to deplete resources for the pathogen (Serrano et al., 2016). Thereupon, we also observed a slight increase of expression of some of these genes at 18 hpi; genes belonging to the photosystem I and II protein complexes and chloroplast’s NHD (Supplementary Table 2). Indeed, this pattern could be related to the pathogen effectors that induce these genes to inhibit CO_2_ intake through disruption of photosystem II, which results in the inhibition of the chloroplastic reactive oxygen burst (de Torres Zabala et al., 2015).

Following the course of the infection, the first signs of activation of the plant defense responses were the upregulation of defense related genes at 6 hpi. Some of the genes were related to recognition of *avirulence genes* (*AVR*), like *LRRs* and *serine/threonine-protein kinases*, which are involved in programmed cell death (PCD) (Romeis, 2001; Liu et al., 2016). Also present in this group were transcription factors that play an important role in cascade signaling of MAPK, like *ethylene-responsive transcription factors* (*ERF*) and *WRKY* 6, 22 and 44, which are necessary in the expression of defense genes (Zhang and Klessig, 2001; Birkenbihl et al., 2017). The activation of these genes was followed by a sharp decrease in expression at 12 hpi, and a steady decline until the final stages of infection.

Even though we observed the expression of pathways involved in HR as mentioned above, we can presume that oomycete effectors are capable of suppressing the host immunity resulting in ETS (Resjö et al., 2019). Also, we observed expression of resistance genes, such as *putative late blight resistance protein homolog R1A-10* that recognizes Avr1 of *P. infestans*, but it is reasonable to consider that the effectors proteins are not being recognized by these *R*-genes or the gene-for-gene interaction is blocked by other effector proteins (Halterman et al., 2010; Yang et al., 2018) because the infection successfully progressed until necrotrophy. Nonetheless, functional analysis is needed to establish the nature of this interaction.

At 12 hpi we found upregulation of genes related to circadian rhythm as well as abiotic stress such as *bHLH* and *MYB1R1* transcription factors, key factors in the circadian clock complex, and *Cytochrome P450* monooxygenases suggesting a regulation of expression caused by an external stimulus. The mediation of defense responses against environmental stress is regulated by the circadian rhythm (Thines et al., 2019). Primarily, the level of salicylic and jasmonic acid, and other plant defense hormones are related with the circadian rhythm. Timely accumulation of these phytohormones is essential to confront the pathogen, either by PTI or ETI (Lankinen et al., 2018). This may explain the early expression of *the pathogenesis-related protein PR-1* and *salicylate carboxymethyl transferase* (SAMT), which are key components in the salicylic acid-mediated disease resistance (Breen et al., 2017). It is presumed that hemibiotrophic pathogens induce SA pathways early at the biotrophic phase, which concur with the expression patterns observed at this time in this pathosystem (Zuluaga et al., 2016).

At 18 hpi, we observed expression of strengthening of the plant cell wall genes, most likely related to an effort by the plant to halt secondary infections (van Schie and Takken, 2014). Nevertheless, cell wall biogenesis can also serve as a source of nutrients for the pathogen. Although the *CesA8* is an essential enzyme for plant cell wall formation, it can also increase susceptibility as demonstrated in previous reports (Miedes et al., 2014; van Schie and Takken, 2014). There was also enrichment of genes related to carbohydrate metabolism, which play a role in plant immunity, acting as elicitors and signaling much like phytohormones (Trouvelot et al., 2014). The sugar transporter *SWEET1*, which is involved in regulation of the sucrose pools in plants was induced in this cluster at 18 hpi, acting as a susceptible factor, as they can be hijacked by the pathogen to access carbohydrate resources for growth (Jiang S. et al., 2020).

The presence of the genes *SWEET1*, *SWEET2*, and *SWEET12* was also detected, with a peak increase in expression at 18 hpi. The overexpression of these genes at this time have been associated with the pathogen manipulation of the host, as the use of sugars like fructose and amino acids are essential resources for a successful colonization, as described in *P. infestans* (Botero et al., 2018). This also indicates that the interaction between *S. betaceum* and *P. betacei* is compatible, as the induction of expression of these genes correlates with a more susceptible interaction (Duan et al., 2020).

Of the functional category for secondary metabolite production, at 24 hpi there was an overall enrichment, in particular of biosynthesis of terpenoid, phenylpropanoid, and lignin, generally active defense pathways against biotic and abiotic stress (Paolinelli-Alfonso et al., 2016). The synthesis of a secondary wall containing lignin helps in the reinforcement of the wall and hinders the entry of the pathogen (Miedes et al., 2014). Likewise, the activation of terpenoid and phenylpropanoid produces phytoalexins, which present antimicrobial properties and accumulate in dying cells (Bell et al., 1986). Nonetheless, the expression of phytoalexins in early infection is related to an incompatible interaction (host resistance) (Bell et al., 1986).

At 72 hpi, we observed a clear response of the plant expression to the pathogen switch: genes related to intracellular signaling and HR are activated. Noteworthy, *ERF*, *putative late blight protein R1-A10* and *R1B-23*, and *senescence-specific cysteine protease SAG12* were expressed, suggesting an induced leaf senescence, triggered by the pathogen to complete the infection cycle (Noh and Amasino, 1999). As infection approached 96 hpi, genes related to these terms continue their induction with addition to senescence related terms and jasmonic acid metabolic process. The jasmonate and ethylene signal pathways are commonly associated with the response to necrotrophic pathogens (Sun, 2017).

## Conclusion

In conclusion, we obtained the first time-series transcriptome of *S. betaceum* with a comprehensive expression profile across infection caused by *P. betacei*. From these data, we observed a close interaction between the host transcriptional response and the hemibiotrophic infection strategy of the pathogen, exhibiting a dynamic defense-related gene response throughout the course of infection. We observed different upregulated genes, related to susceptibility and resistance, that elucidate the continuous response in this compatible interaction: from the recognition of the pathogen and the activation of defense related pathways to the final stages of infection with the expression of genes associated with cell death. We hypothesized the nature of this interaction as ETS, with a reprogramming of the host transcription caused by the pathogen for essential resources to aid in its colonization. Further analysis with resistant cultivars can be useful to understand the molecular mechanisms underlying resistance in tree tomato.

## Data Availability Statement

The transcriptome assembly and the raw reads are accessible at NCBI BioProject database with the submission accession PRJNA743564.

## Author Contributions

SR, AB, and NG-P planned and designed the research. NG-P, DBa, and MCB performed the experiments. DBa, DBo, JD, AB, and SR analyzed the data. DBa, JD, MC, and SR wrote the manuscript. All authors contributed to the article and approved the submitted version.

## Conflict of Interest

The authors declare that the research was conducted in the absence of any commercial or financial relationships that could be construed as a potential conflict of interest.

## Publisher’s Note

All claims expressed in this article are solely those of the authors and do not necessarily represent those of their affiliated organizations, or those of the publisher, the editors and the reviewers. Any product that may be evaluated in this article, or claim that may be made by its manufacturer, is not guaranteed or endorsed by the publisher.

## Footnotes

1
http://www.ncbi.nlm.nih.gov/VecScreen/UniVec.html

2
https://transdecoder.github.io
