# Supplementary material for: Comprehensive Time-Series Analysis of the Gene Expression Profile in a Susceptible Cultivar of Tree Tomato (Solanum betaceum) During the Infection of Phytophthora betacei
Source: Front Plant Sci. 2021 Oct 21;12:730251. doi: 10.3389/fpls.2021.730251 (PMC8567061; doi:10.3389/fpls.2021.730251)
Supplement: Supplementary file 1 [file Data_Sheet_1.docx]

**Comprehensive time-series analysis of the gene expression profile in a susceptible cultivar of tree tomato (*Solanum betaceum*) during the infection of *Phytophthora betacei***

The following Supporting Information is available for this article:


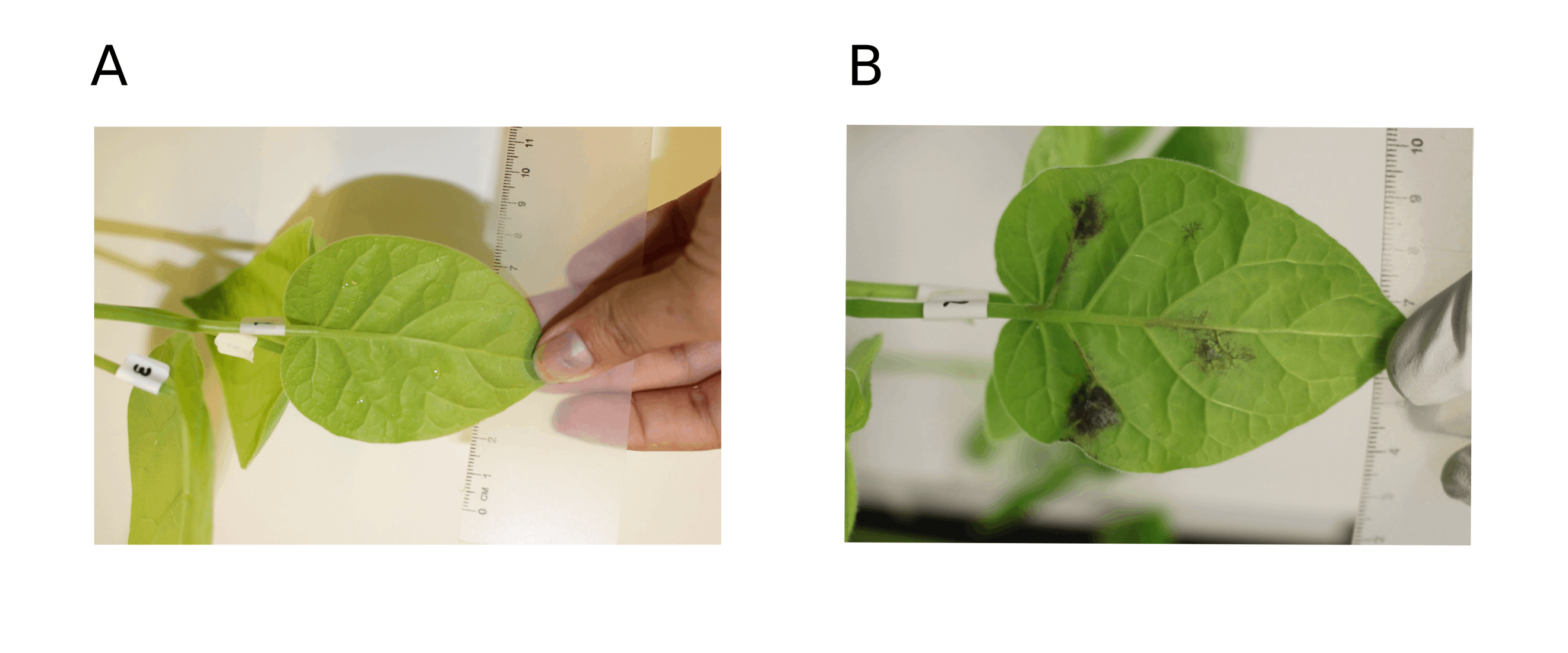


**Fig. S1** Development of lesions for tree tomato cultivar Comun at A) 1 day and B) 6 days post inoculation with a *Phytophthora betacei* isolate.


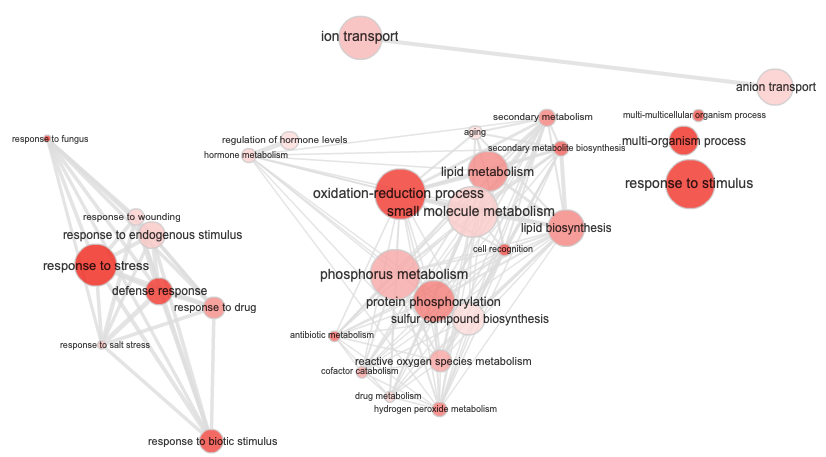

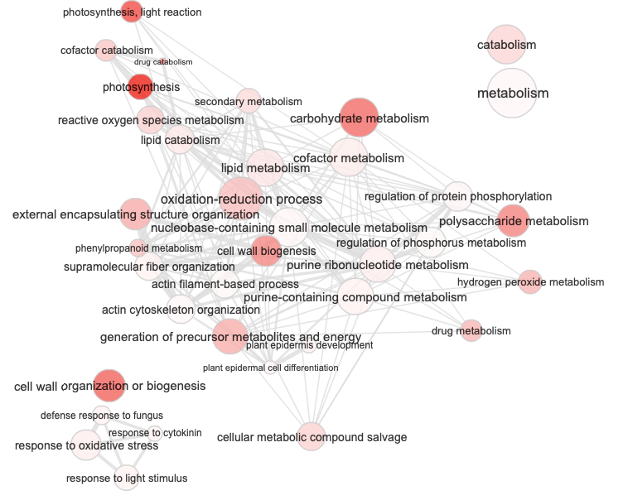


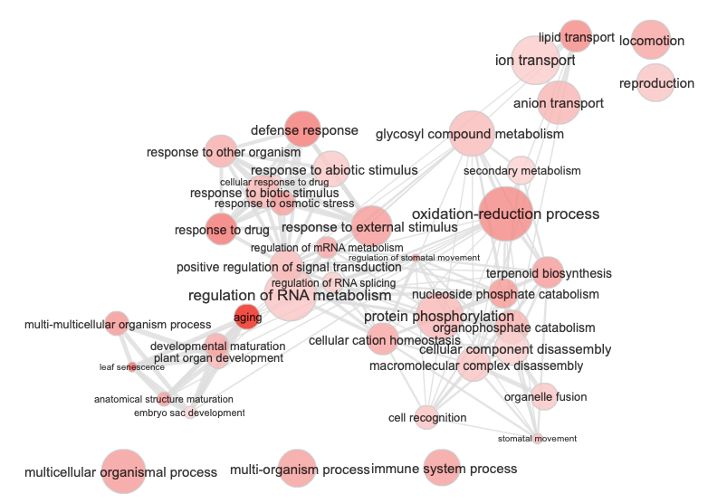

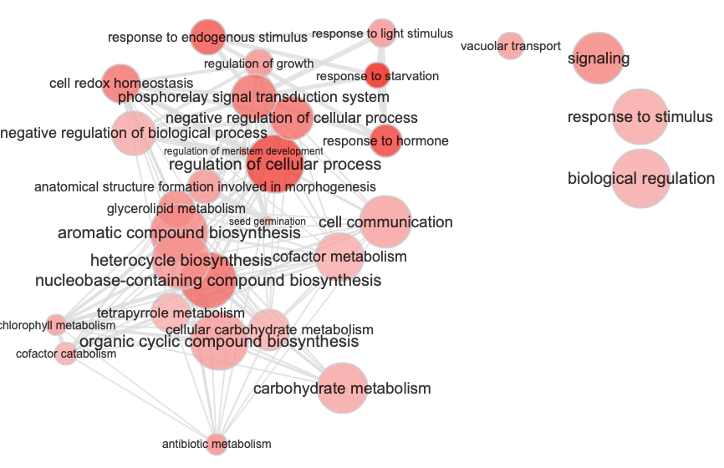


**Fig. S2** Enriched Gene Ontology Terms from differentially expressed genes (2-fold, *P* < 0.001) comparing uninoculated samples (0 hpi), early (6, 12, 18 and 24 hpi) and late times of infection (72 and 96 hpi). a) upregulated biotrophic terms (up left) b) down regulated biotrophic genes (up right) c) up regulated necrotrophic genes (down left) d) down regulated necrotrophic genes (down right).


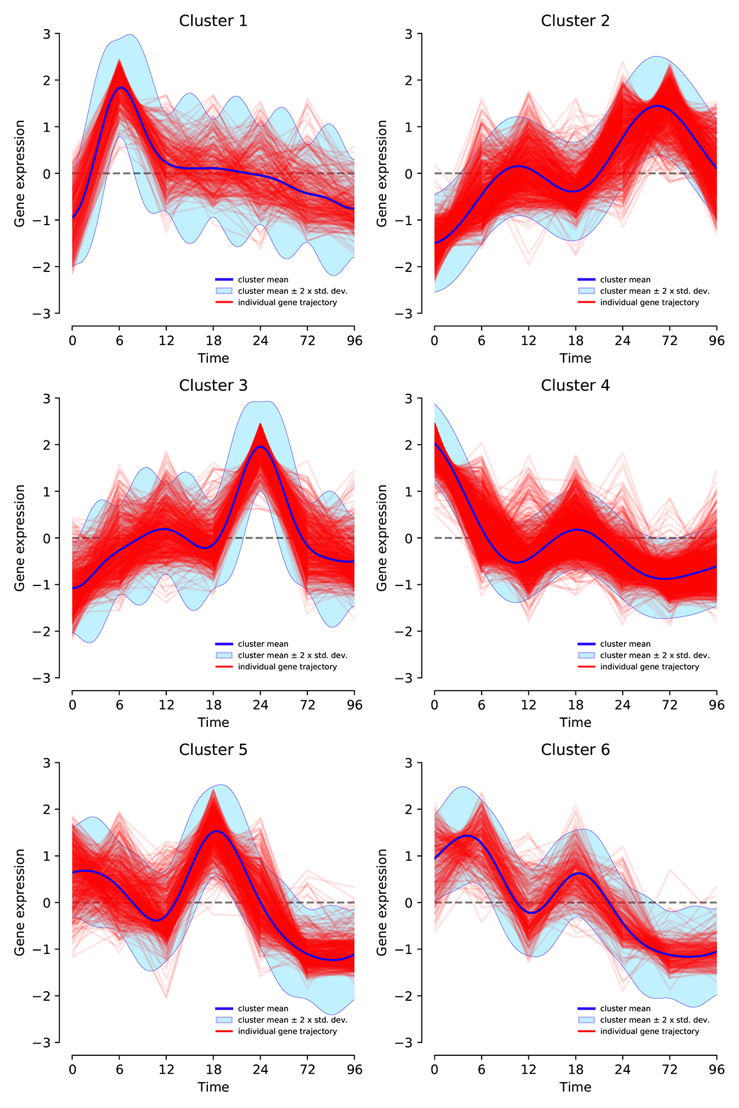

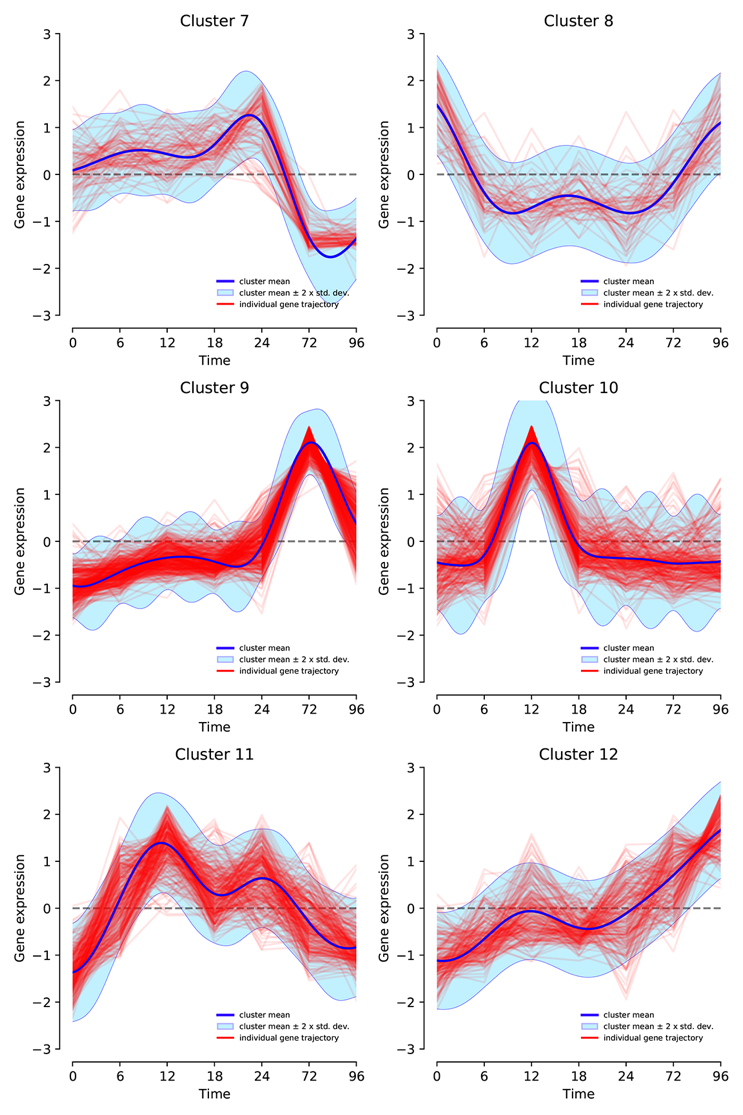


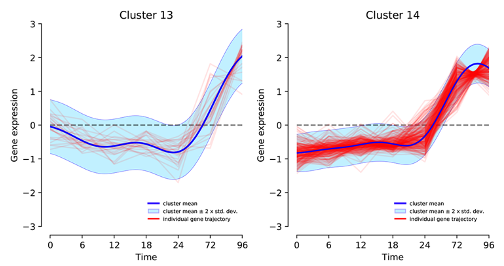

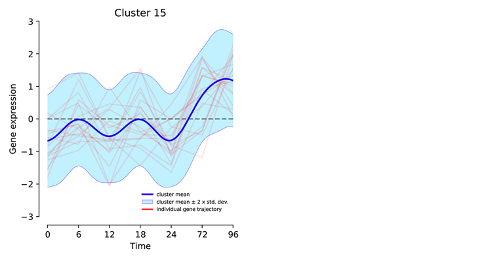


**Fig. S3** Differentially expressed genes (4-fold, *P* < 0.001) for all infection times (0, 6, 12, 18, 24, 72, 96 hours post inoculation) were clustered based on their expression profile. 15 different clusters based on Maximum a posteriori method were produced, and matched the number of each group in the heatmap.


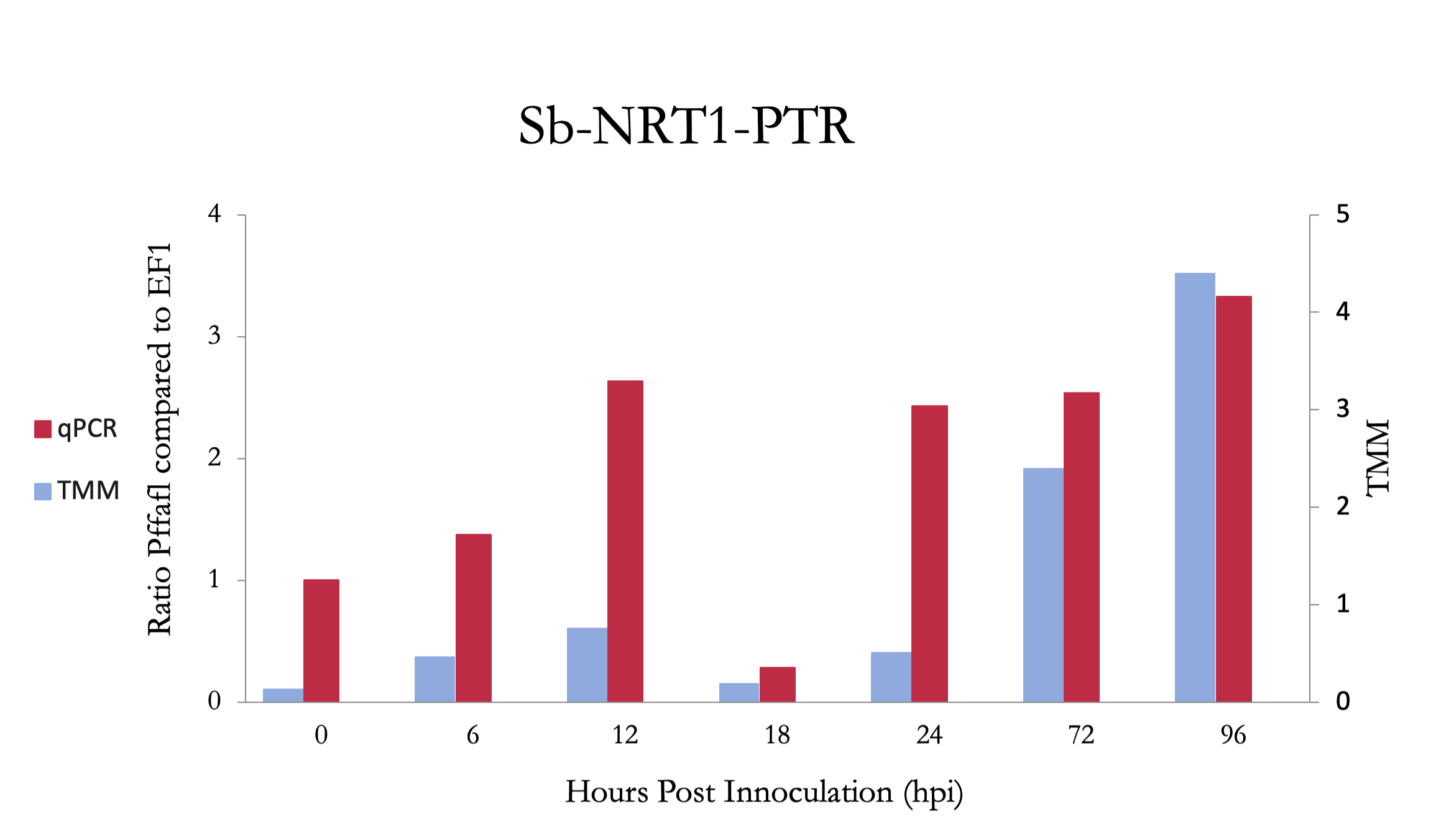

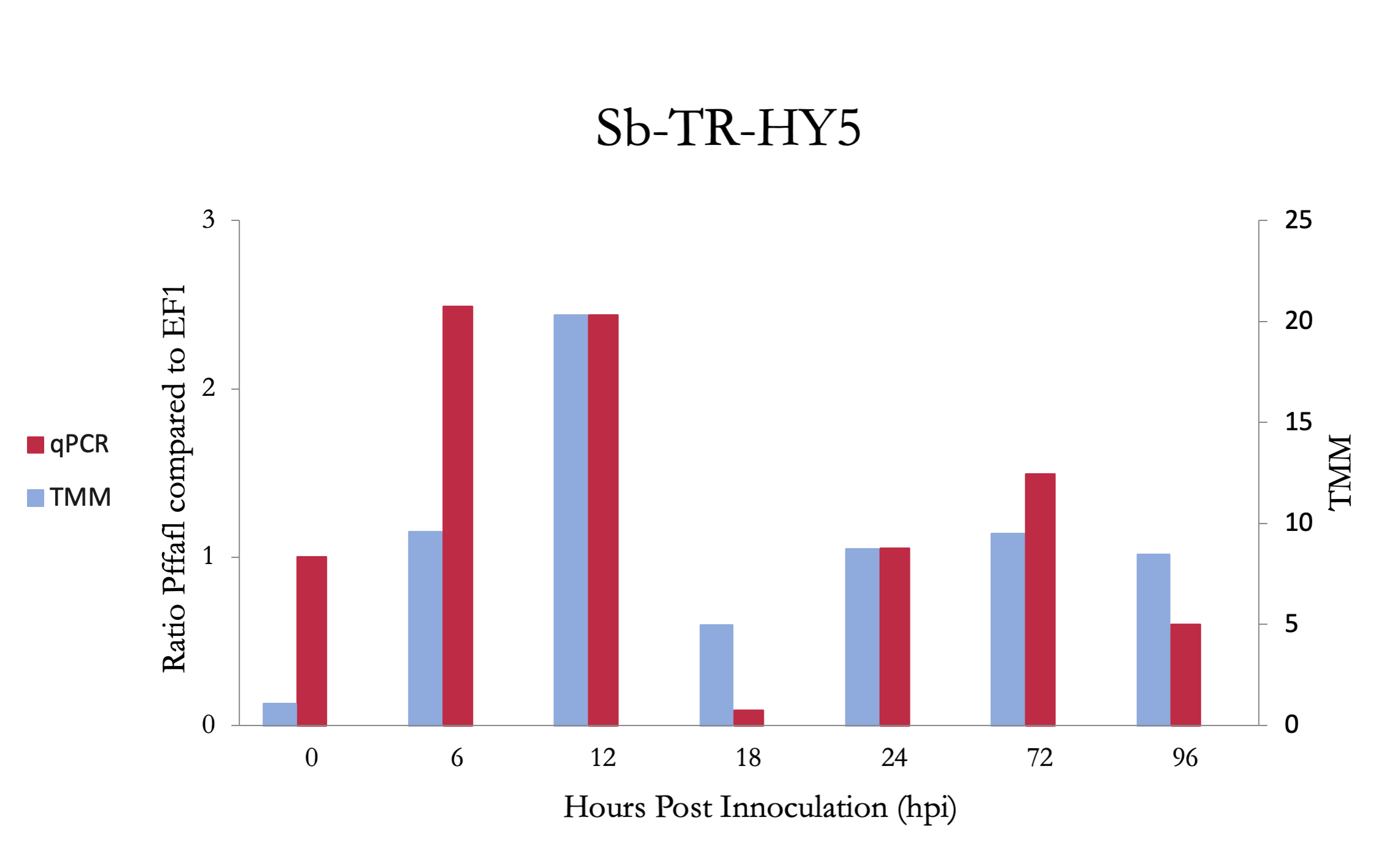

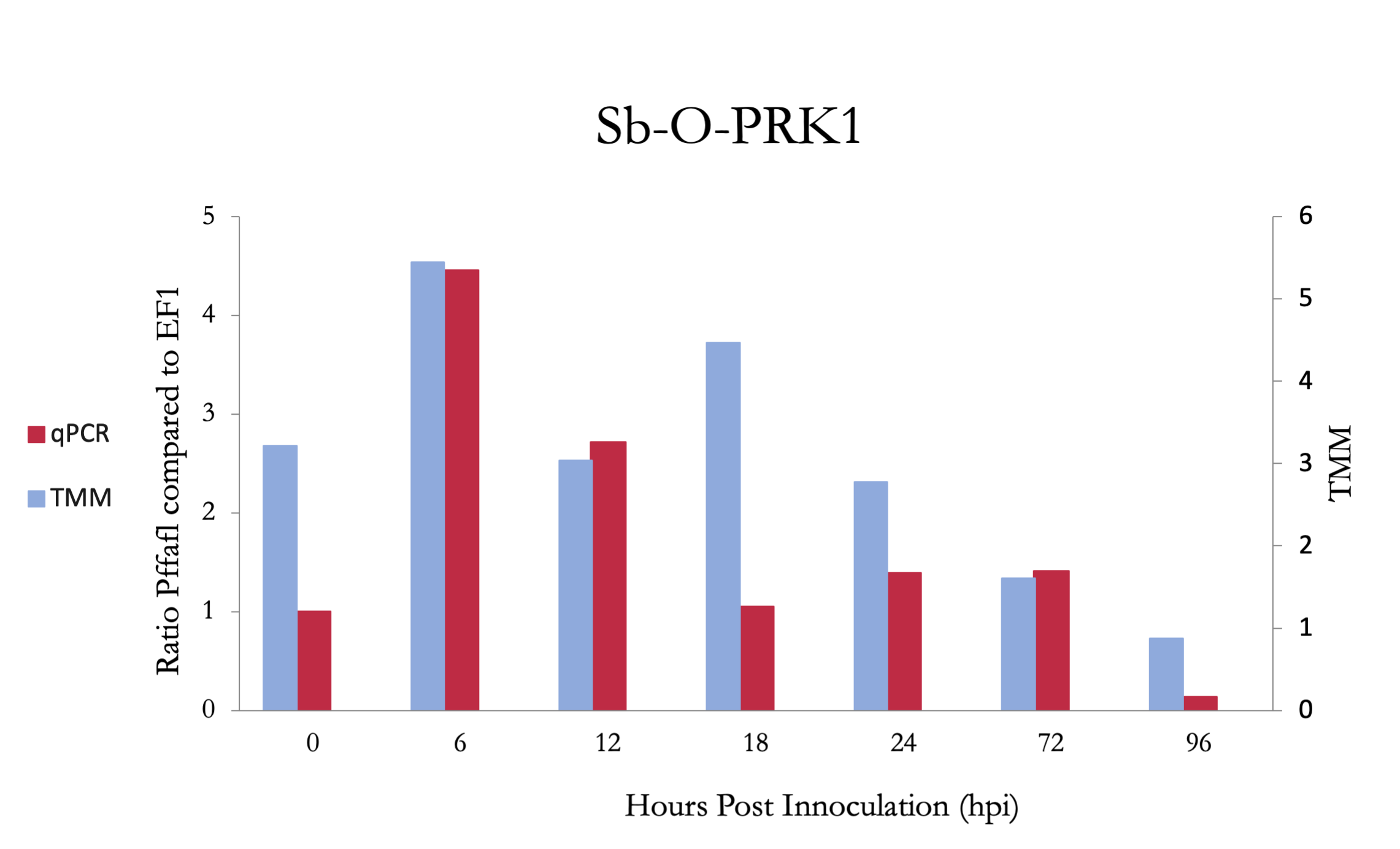

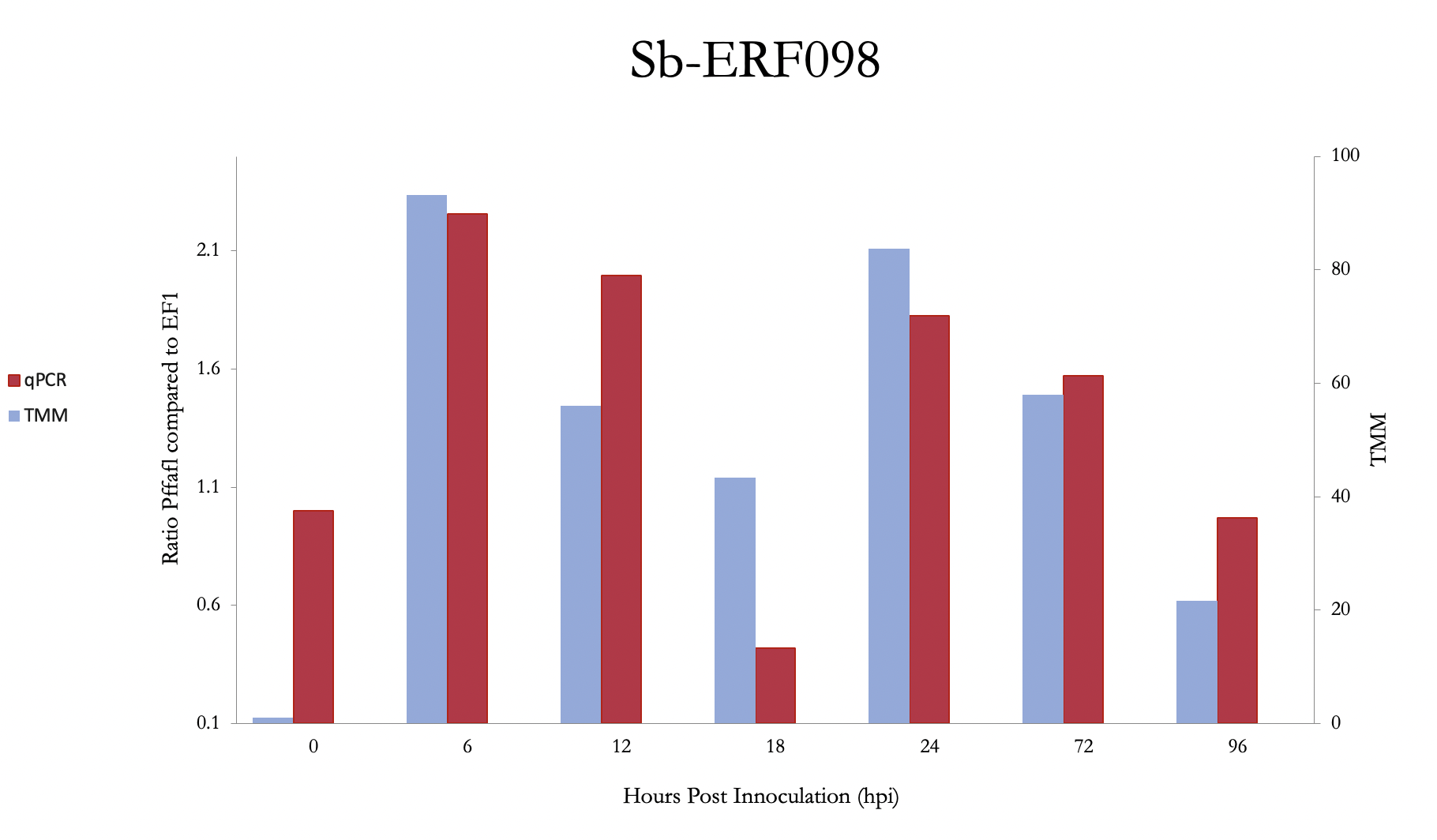


**
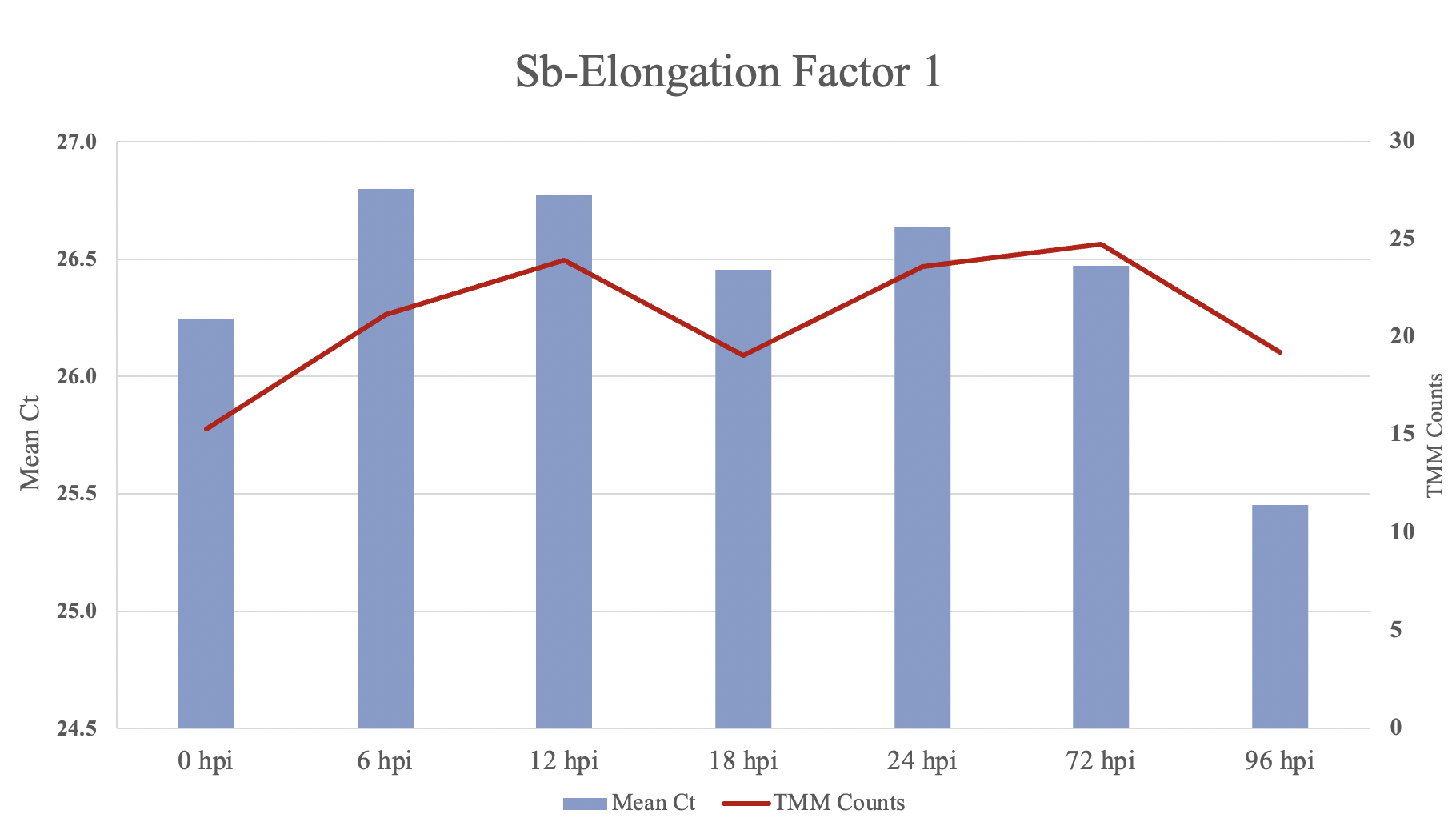
**

**Fig. S4** RT-qPCR results for RNA-seq validation. Red bars represent Pfaffl Ratio of compared expression between normalized gene (*Elongation Factor 1*) and four different genes evaluated. Blue bars represent TMM values for each gene at each of the time points. Genes evaluated were: NRT1/ PTR FAMILY like (Sb-NRT1-PTR), *Transcription Factor HY5* (Sb-TR-HY5), *Probable Receptor-like Protein Kinase* (Sb-O-PRK1) and *Ethylene-responsive Transcription Factor* ERF098-like (SB-ERF098). Ct median and TMM values for the Housekeeping gene *Elongation Factor 1* is shown.

**
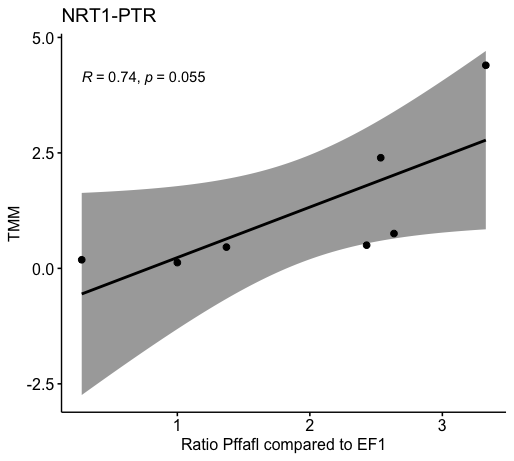
** **
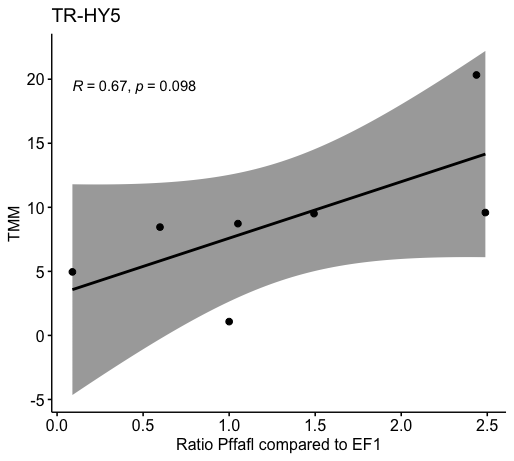
**

**
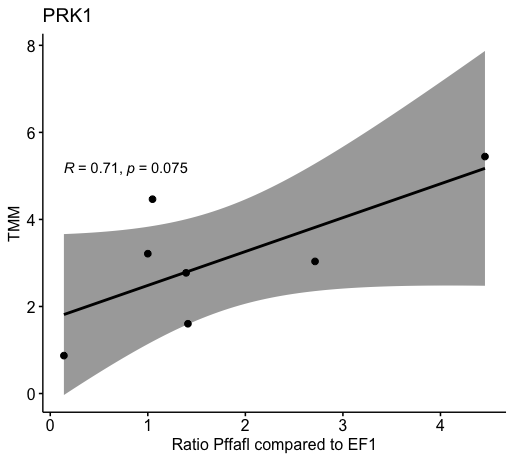

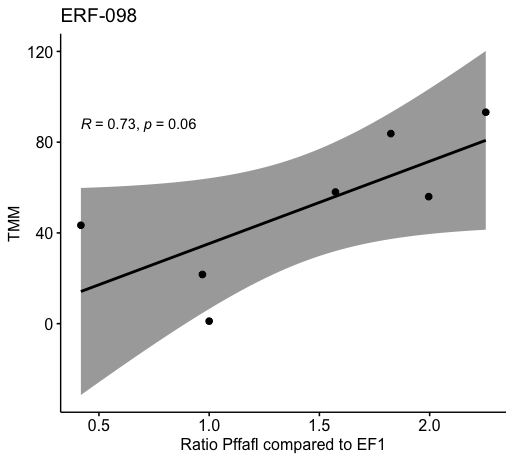
**

**Fig. S5** Pearson’s Correlation results between Pffafl Ratio and TMM values for the four genes selected for RNA-seq validation. Genes evaluated were: NRT1/ PTR FAMILY like (Sb-NRT1-PTR), *Transcription Factor HY5* (Sb-TR-HY5), *Probable Receptor-like Protein Kinase* (Sb-O-PRK1) and *Ethylene-responsive Transcription Factor* ERF098-like (SB-ERF098).

**Table S1** List of Enriched Gene Ontology terms of Biological Process for all 15 clusters based on expression profiles throughout time series.

**Table S2** List of annotated genes belonging to the 15 clusters based on expression profiles throughout time series.
